# *CHEK2* Mutations Affecting Kinase Activity Together With Mutations in *TP53* Indicate a Functional Pathway Associated with Resistance to Epirubicin in Primary Breast Cancer

**DOI:** 10.1371/journal.pone.0003062

**Published:** 2008-08-26

**Authors:** Ranjan Chrisanthar, Stian Knappskog, Erik Løkkevik, Gun Anker, Bjørn Østenstad, Steinar Lundgren, Elisabet O. Berge, Terje Risberg, Ingvil Mjaaland, Lovise Mæhle, Lars Fredrik Engebretsen, Johan Richard Lillehaug, Per Eystein Lønning

**Affiliations:** 1 Section of Oncology, Institute of Medicine, University of Bergen, Bergen, Norway; 2 Department of Oncology, Haukeland University Hospital, Bergen, Norway; 3 Department of Molecular Biology, University of Bergen, Bergen, Norway; 4 Department of Oncology, The Norwegian Radium Hospital, Rikshospitalet University Hospital, Oslo, Norway; 5 Department of Oncology, Ullevaal University Hospital, Oslo, Norway; 6 Department of Oncology, St. Olav University Hospital, Trondheim, Norway; 7 Department of Oncology, University Hospital of Northern Norway and Institute of Clinical Medicine, University of Tromsø, Tromsø, Norway; 8 Division of Hematology and Oncology, Stavanger University Hospital, Stavanger, Norway; 9 Department of Medical Genetics, Rikshospitalet University Hospital, Oslo, Norway; 10 Center for Medical Genetics and Molecular Medicine, Haukeland University Hospital, Bergen, Norway; 11 Norwegian University of Science and Technology, Department of Cancer Research and Molecular Medicine, Trondheim, Norway; Northwestern University, United States of America

## Abstract

**Background:**

Chemoresistance is the main obstacle to cure in most malignant diseases. Anthracyclines are among the main drugs used for breast cancer therapy and in many other malignant conditions. Single parameter analysis or global gene expression profiles have failed to identify mechanisms causing *in vivo* resistance to anthracyclines. While we previously found *TP53* mutations in the L2/L3 domains to be associated with drug resistance, some tumors harboring wild-type *TP53* were also therapy resistant. The aim of this study was; 1) To explore alterations in the *TP53* gene with respect to resistance to a regular dose epirubicin regimen (90 mg/m^2^ every 3 week) in patients with primary, locally advanced breast cancer; 2) Identify critical mechanisms activating p53 in response to DNA damage in breast cancer; 3) Evaluate *in vitro* function of Chk2 and p14 proteins corresponding to identified mutations in the *CHEK2* and *p14^(ARF)^* genes; and 4) Explore potential *CHEK2* or *p14^(ARF)^* germline mutations with respect to family cancer incidence.

**Methods and Findings:**

Snap-frozen biopsies from 109 patients collected prior to epirubicin (as preoperative therapy were investigated for *TP53, CHEK2* and *p14^(ARF)^* mutations by sequencing the coding region and *p14^(ARF)^* promoter methylations. *TP5*3 mutastions were associated with chemoresistance, defined as progressive disease on therapy (*p* = 0.0358; *p* = 0.0136 for mutations affecting p53 loop domains L2/L3). Germline *CHEK2* mutations (n = 3) were associated with therapy resistance (*p* = 0.0226). Combined, mutations affecting either *CHEK2* or *TP53* strongly predicted therapy resistance (*p* = 0.0101; *TP53* mutations restricted to the L2/L3 domains: *p* = 0.0032). Two patients progressing on therapy harbored the *CHEK2* mutation, Arg95Ter, completely abrogating Chk2 protein dimerization and kinase activity. One patient (Epi132) revealed family cancer occurrence resembling families harboring *CHEK2* mutations in general, the other patient (epi203) was non-conclusive. No mutation or promoter hypermethylation in *p14^(ARF)^* were detected.

**Conclusion:**

This study is the first reporting an association between *CHEK2* mutations and therapy resistance in human cancers and to document mutations in two genes acting direct up/down-stream to each other to cause therapy failure, emphasizing the need to investigate functional cascades in future studies.

## Introduction

Chemoresistance is the main obstacle to cure in most malignancies, including breast cancer. While adjuvant chemotherapy may reduce the hazard rate of relapse by about one third in breast cancer patients [1], the majority among patients harboring micro- metastases are not cured by today's standards. Considering patients harboring distant metastases, resistance and therapy failure inevitably occurs, in general over a time period of less than one year for each individual regimen [2].

Despite extensive experimental research [3], little data are available considering chemoresistance *in vivo*. For anthracycline therapy in breast cancer, topoisomerase-II amplifications have been associated with a dose-responsiveness different from what is observed in non-amplified tumors 4, 5. Several studies have tried to generate “prediction profiles” based on gene expression microarrays [6], [7], [8], however, none of the different profiles generated expressed a sensitivity suitable for clinical applications, or have been successfully reproduced by others (see references to original works in [9] and [10]).

p53 (the protein encoded by the *TP53* gene) plays a key role in executing DNA-damage induced apoptosis and growth arrest [11]. Previously, our group reported mutations in the zink-binding domains L2 (codons 163–195) and L3 (codons 236–251) of p53 critical to DNA binding [12] to be associated with but not fully predictive for resistance to chemotherapy with a low-dose weekly anthracycline [13] or a mitomycin plus 5-fluoro-uracil containing [14] regimen. Similar findings were reported by another group [15]. In contrast, others reported *TP53* mutations to predict sensitivity to a dose-dense epirubicin-cyclophosphamide regimen [16].

The finding that some tumors harboring wild-type *TP53* may be resistant to anthracycline therapy lead us to postulate that other genes involved in the p53 pathway could be mutated in these tumors [3]. p53 is activated by post-translational modifications, and the protein is phosphorylated at multiple amino acids [17]. Phosphorylation at Ser 20 (Ser 23 in mice) by the Chk2 protein (coded by the *CHEK2* gene) in response to DNA damage activates p53 by inhibiting binding to, and deactivation by, the MDM2 (Mouse Minute 2 homolog; HDM2) protein [18], [19], [20]. While experimental studies have suggested a critical role of Chk2 in activating p53 apoptotic response to genotoxic stress [21], [22], others claim Chk2 to be dispensable for p53 activation with respect to apoptosis as well as growth arrest [23]. Following an initial report of a *CHEK2* germline mutation in a family filling the characteristics of a Li-Fraumeni syndrome (LFS) [24], recent papers have suggested germline mutations in *CHEK2* to be associated with a moderately increased risk of breast and colon cancers (see references in [25]). Recently, we discovered a somatic, nonsense *CHEK2* mutation in a single patient expressing resistance to doxorubicin low dose therapy [26].

A second mechanism of p53 activation is through p14^(ARF)^ (p19 in mice) function. p14^(ARF)^ does not phosphorylate p53, but inhibits MDM2 dependent p53 degradation through direct MDM2 binding. While p14^(ARF)^-mediated p53 activation has been linked to oncogene-induced p53 activation and, in general, considered not involved in response to DNA damage (see references in [27]), p14^(ARF)^ may be activated through the E2F1/retinoblastoma pathway [28]. Importantly, two recent studies revealed lack of p19 (mouse homologue of human p14^(ARF)^) function in mice to inhibit p53 tumor suppressor function in response to ionizing radiation as well as DNA damaging agents [29], [30].

The aim of this study was 1) to explore alterations in the *TP53* gene with respect to resistance to a regular dose epirubicin regimen (90 mg/m^2^ body surface every 3 week) in patient with primary, locally advanced, breast cancer; 2) To explore defects in potential mechanisms activating p53 in response to DNA damage in breast cancer as a cause of drug resistance in wild-type tumors. To do so, we sequenced the complete coding regions for the *CHEK2* and *p14^(ARF)^* genes and analyzed for *p14^(ARF)^* promoter hypermetylations; 3) Evaluate *in vitro* function of potential Chk2 and p14^(ARF)^ protein translates corresponding to identified mutations in the *CHEK2* and *p14^(ARF)^* genes; 4) Identify potential *TP53, CHEK2* and *p14^(ARF)^* mutations to be germline, explore the incidence of different cancers among affected relatives with respect to specific mutations. By comparing *in vitro* characteristics of specific mutations to drug sensitivity and family cancer risk syndromes, this may add to our understanding of the importance of these gene cascades executing response to DNA damage versus tumor suppression activity.

Analyzing tumor samples from a total of 109 primary locally advanced breast cancer patients treated with epirubicin 90mg/3 weekly, we found *TP53* mutations affecting the L2/L3 domains or protein dimerization, as well as non-functional *CHEK2* mutations abrogating dimerization and phosphorylation, to be associated with therapy resistance; no mutation or promoter hypermethylations of the *p14^(ARF)^* gene was discovered. Our findings suggest a critical role for Chk2 with respect to DNA-damage-dependent p53 activation and resistance to anthracycline therapy in human breast cancer.

## Materials and Methods

### Patients

A total of 223 patients with locally advanced non-inflammatory breast cancer (T3-4 and/or N2) were randomly allocated to primary treatment either with epirubicin 90 mg/m^2^ or paclitaxel 200 mg/m^2^. The primary aim of the study was identification of markers predicting drug resistance to the regimens. Thus, the reason for randomizing patients was not for effect comparison, but to achieve similar patient cohorts in the two arms. Based on the findings of a clinical lack of cross-resistance between anthracyclines and taxane therapies in breast cancer [31], we hypothesized the mechanisms of resistance to be different between the two compounds. While the analysis of tumor samples from the paclitaxel is ongoing, we here report our findings from the patients allocated to the epirubicin arm.

The epirubicin arm included a total of 109 patients (age 28 to 70 years, median 51 years). Two patients were analyzed for gene mutations but omitted from statistical analysis as protocol violators; histopathological examination revealed one patient (Epi089) to harbor a sarcomatoid tumor, while one patient Epi232 was erroneously enrolled with stage II disease.

The study protocol was approved by the Regional Ethical Committee (Norwegian Health Region III), including formal Biobank registration in accordance to Norwegian law. The study and protocol is registered under the Norwegian Social Science Data services ((www.nsd/uib/personvern/database/), University of Bergen project no 16297 and Helse Bergen project no 13025). Each patient gave written informed consent.

### Tissue Sampling

Before commencing chemotherapy, each patient had an incisional tumor biopsy as described previously [14]. All tissue samples were snap-frozen immediately on removal in the theatre.

### Treatment Regime and Staging

Primary treatment consisted of epirubicin (90 mg/m^2^) administered as a 3-weekly schedule. Treatment was scheduled for four cycles unless progression occurred at an earlier stage. Clinical response was assessed before each treatment cycle, and the final response evaluated 3 weeks after the 4^th^ cycle for overall response classification. Because the protocol was implemented by October 1997 with patients enrolled between November 1997 and December 2003, responses were consistently graded by the UICC system [32] and not the more recently implemented “RECIST” criteria [33]. Thus, responses were classified as CR (Complete Response, complete disappearance of all tumor lesions), PR (Partial Response, reduction ≥50% in the sum of all tumor lesions, calculated for each as the product of the largest diameter and the one perpendicular to it), PD (Progressive Disease, increase in the diameter product of any individual tumor lesion by ≥25%), and SD (Stable Disease, anything between PR and PD). To analyze for the predictive value of the different parameters, similar to our previous studies [13], [14] we compared PD tumors (non responders) with the combined group of tumors classified as SD/PR/CR (responders); the reason for this approach is discussed in detail elsewhere [34]. Median follow-up time was defined from patient inclusion in the study up to October 31, 2006. Deaths attributable to causes other than breast cancer were treated as censored observations.

All patient records were subject to central audit for response classification (by E.L., B.Ø. and P.E.L.). Response classifications were completed and approved without any knowledge about result from laboratory analysis.

### RNA Purification

Total RNA was purified by Trizol (Life Technologies, Inc.) extraction from snap-frozen tissue samples according to manufacturer's instructions. After extraction, the RNA was dissolved in 100 µl of DEPC treated ddH_2_O. cDNA was synthesized by reverse transcription using Transcriptor reverse transcriptase (Roche), according to the manufacturer's protocol.

### DNA Purification

Genomic DNA from tumor biopsies and blood lymphocytes was isolated using QIAamp DNA Mini kit (Qiagen, Chatsworth, CA) according to the manufacturer's protocol.

### Mutation Analysis

All mutational analysis was performed blinded to clinical data. Mutations in *TP53*, *CHEK2* and *p14^(ARF)^* genes were analyzed by PCR (or nested PCR) amplification and sequencing of PCR product, or by cloning of PCR products and sequencing of the resulting plasmids (all primers described in Table 1). Cloning was performed using the TOPO TA Cloning kit (Invitrogen). Sequencing of clones was performed until at least 10 different sequences covered all parts of the *CHEK2* coding sequence. DNA sequencing was carried out directly on 1 µl PCR product or plasmid using Big Dye terminator mix (Applied Biosystems). Capillary gel electrophoresis, data collection, and sequence analysis were done on an automated DNA sequencer (ABI 3700). When a mutation was detected, the relevant exon was amplified by PCR from genomic tumor DNA and DNA from blood lymphocytes and sequenced for verification and germline detection. (Primers described in Table 1).

**Table 1:** PCR primers for amplification and sequencing of cDNA

	*TP53*	Orientation	*CHEK2*	Orientation
1.Round	p53 ns2: 5′-gac act ttg cgt tcg ggc	Forward	chk2s1: 5′-atg tct cgg gag tcg gat g	Forward
	p53 nas2: 5′-ctt gtt cag tgg agc ccc g	Reverse	chk2as1: 5′-acc acg gag ttc aca aca cag	Reverse
2.Round	p53 frag1s: 5′-gac acg ctt ccc tgg att ggc	Forward	chk2s3: 5′-ctc ctc tac cag cac gat gc	Forward
	P53 frag4as: 5′-cgc aca cct att gca agc aag gg	Reverse	chk2as2: 5′-aga acc tgg ggt aga gct gtg	Reverse
Sequencing primers	p53 frag3s: 5′-tgg ccc ctc ctc agc atc tta	Forward	chk2s3: 5′-ctc ctc tac cag cac gat gc	Forward
	p53 frag2as: 5′-ggt aca gtc aga gcc aac ctc	Reverse	chk2-7F: 5′-atc atc ctt gca tca tca ag	Forward
			chk2-7R: 5′-atc aat tcc aaa aca ata taa taa tc	Reverse
	*p14*			
1.Round	p14 f2: 5′-cggcgagaacatggtgcg	Forward		
	p14 r2: 5′-ttcccgaggtttctcagagcc	Reverse		
2.Round	p14 f2: 5′-cggcgagaacatggtgcg	Forward		
	p14 nest r: 5′-tct ctg gtt ctt tca atc g	Reverse		
Sequencing primers	p14 nest r: 5′-tct ctg gtt ctt tca atc g	Reverse		
PCR primers for amplification and sequencing of genomic DNA				
Exon 1			Chk2 ex1F 5′-gtc ttg tgc ctt gaa act c	Forward
			Chk2 ex1R 5′-cca cct ggt aat aca act tt	Reverse
Exon 5	p53 ex5r 5′-ctg ttc act tgt gcc ctg act tt	Forward		
	p53 ex5r 5′-gga atc aga ggc ctg ggg ac	Reverse		
Exon 6	p53 ex6f 5′-gac gac agg gct ggt tgc	Forward		
	p53 ex6r 5′-gcc act gac aac cac cct taa	Reverse		
Exon 7	p53 ex7f 5′-gct tgc cac agg tct ccc	Forward		
	p53 ex7r 5′-gca gag gct ggg gca ca	Reverse		
Exon 8	p53 ex8f 5′-gga cct gat ttc ctt act gcc	Forward		
	p53 ex8r 5′-gtg aat ctg agg cat aac tg	Reverse		
Exon 9	p53 ex9f 5′-caa gaa gcg gtg gag gag a	Forward	Chk2 ex9F 5′-acg gct tac ggt ttc acc	Forward
	p53 ex9r 5′-aac ggc att ttg agt gtt aga c	Reverse	Chk2 ex9R 5′-caa gaa tct aca gga ata gcc	Reverse
Exon 10	p53 ex10f 5′-ctc ccc ctc ctc tgt tgc tg	Forward		
	p53 ex10r 5′-aag gca gga tga gaa tgg aat c	Reverse		
Sequencing primers	Either forward or reverse primer were used		Either forward or reverse primer were used	
*p14* Methylation spesific primers				
Methylated	p14_met s 5′-gtg tta aag ggc ggc gta gc	Forward		
	p14_met as 5′-aaa acc ctc act cgc gac ga	Reverse		
Unmethylated	p14_umet s 5′-ttt ttg gtg tta aag ggt ggt gta gt	Forward		
	p14_umet as 5′-cac aaa aac cct cac tca caa caa	Reverse		

### Loss of Heterozygosity (LOH)

Loss of heterozygosity (LOH) in tumors with mutations in *CHEK2* was assessed using the microsatellite marker, D22S275, which maps to intron 4 of *CHEK2*. LOH in tumors with mutation in *TP53* was assessed using two markers, one variable number tandem repeat in intron 1 [35] and a CA repeat close to the *TP53* gene [36]. Fluorescently end-labeled primers were used in the PCR, and the PCR products were analyzed on an ABI 3700. LOH was evaluated by comparing the allele peak-height ratios from blood DNA and tumor DNA. A sample was scored as having AI (Allelic Imbalance) when a reduction in peak height of one allele in tumor sample was at least 18% compared with that of blood DNA from the same patient [37].

### Analysis of *p14^(ARF)^* promoter methylation

Genomic DNA was subjected to bisulphate conversion using the CpGenome DNA Modification Kit (Intergen) according to the manufacturer's protocol. Both the unmethylated- and methylated-specific PCRs were performed in 50 µl reaction mixes containing 2.5 U AmpliTaq Gold DNA Polymerase (Applied Biosystems), 1× PCR buffer, 1.5 mM MgCl_2_, 0.1 mM of each deoxynucleotide triphosphate, 0.2 µM of each primer (Table 1) and 2 µl of modified genomic DNA. Thermocycling conditions for both the unmethylated- and methylated-specific PCRs were an initial step of 5 minutes at 95°C followed by 35 cycles of 30 sec. at 94°C, 30 sec. at 60.5°C and 60 sec. at 72°C before a final elongation step at 72°C for 7 min.

### Chk2 Dimerisation

Chk2 mutant's ability to form dimers with the wild-type protein was investigated by immunoprecipitation. U-2-OS cells were co-transfected with expression vectors expressing wild-type Chk2 with N-terminal Xpr-tag (pcDNA4/HisMax, Invitrogen) and mutated Chk2 forms with C-terminal V5-tag (pcDNA3.1/V5-His, Invitrogen). Transfection was performed using FuGene 6.0 transfection reagent (Roche) according to the manufacturer's instructions. Cells were harvested in lysisbuffer (50 mM TrisHCl pH 8.0, 150 mM NaCl, 0.5% NP40, 5 mM EDTA pH 8.0) 48 hours after transfection. An aliquote of the cell lysate was harvested for subsequent Chk2-mutant-V5 transfection verification. Samples were further incubated with A/G Pluss Agarose beads (Santa Cruz Biotechnology) at 4°C for 25 minutes before the beads were removed by centrifugation at 5000g for 4 minutes and the samples were incubated with 1.5 µg anti-V5 (Invitrogen) at 4°C for 90 minutes. Fresh A/G Pluss Agarose beads were added and the samples were incubated for another 90 minutes at 4°C. The beads were washed three times with 1×PBS, before being separated on a 10% polyacrylamide gel and blotted on to a nitrocellulose membrane. Chk2-wild-type-Xpr co-precipitated with Chk2-mutant-V5 was detected through incubations with anti-Xpr antibody (Invitrogen), HRP-conjugated secondary antibody and ECL detection reagent (GE Healthcare).

### Kinase Activity

Chk2 mutant's ability to function as kinases was investigated through an *in vitro* kinase assay. The V5 expression vectors used for the dimerisation study were also used to express Chk2 mutants in the kinase assay. U-2-OS cells were transfected using the FuGene 6.0 transfection reagent (Roche) according to the manufacturer's instructions. Cells were then incubated at 37°C in 5% CO_2_ and humidified atmosphere. After 24 hours doxorubicin (Nycomed Pharma) was added to the media to a final concentration of 50ng/ml and the cells were further incubated for 24 hours before harvest. 75 cm^2^ of 90% confluent cells were harvested in 500 µl lysis buffer (50 mM HEPES, 150 mM NaCl, 10% glycerol, 0.5% Triton X-100, 2 mM MgCl_2_, 5 mM EDTA), and the cytosol was incubated for 90 minutes at 4°C with 50 µl 50% Glutathione Sepharose beads (Amersham Biosciences) linked to anti-V5 antibody (Invitrogen). The beads were then washed twice with lysisbuffer containing 500 mM NaCl and twice with kinase assay buffer (50 mM HEPES, 10 mM MgCl_2_, 5 mM MnCl_2_, 2.5 mM EGTA). The beads received 30 µl kinase assay buffer with 7.5 µM cold ATP, 10 µCi^ 32^P-gamma-ATP (GE Healthcare) and 2 µg isolated Cdc25C peptide, and was incubated at 30°C for 30 minutes. Samples were separated on a 12.5% polyacrylamide gel and blotted on to a nitrocellulose membrane. A radiosensitive imaging plate was exposed to the membrane and the plate was read in a FLA200 imager (Fuji).

The kinase assay described above was also used to determine the Chk2 mutants' kinase activity after co-transfection of each Chk2 mutant and wild-type Chk2 in equal amounts.

### Statistical Analysis

Statistical analysis was performed using the Primer of Biostatistics system, version 5.0 [38]. The differences in the distribution of *TP53* and *CHEK2* mutations among patients revealing a PD and the responders were analyzed with use of Fisher's exact test. P-values are reported as accumulated two-sided. Because of the limited time of the follow-up, no formal statistical assessment of overall survival was performed. Relapse-free survival was analyzed by the log-rank test. Details regarding outcome in individual patients with mutations are shown in Table 2 and 3 to make them available to the reader.

**Table 2:** Characteristics of *TP53* mutants found and clinical data

Patient	Age (Yrs)	Clinical response	Codon	Exon	Nukleotide change^1^	Amino acid change	LOH	deleted or inserted sequence	Structural domain	Protein domain	Affecting L2/L3 domain	Predicted mutation	Structure based prediction ^2^	Frequency in database^a^	EReceptor	PReceptor	T	N	M	Relapse-free survivalˆ	Site of Relapse®	Overall survival*
Epi 071	46	CR	175	5	CGC→C**A**C	Arg→His	AI		L2	DNA binding	Yes	missense	non-functional	4.9 (4.1)	Negative	Negative	3	0	0	F72		A72
Epi 220	56	PR	163	5	TAC→T**G**C	Tyr→Cys	ND		S4/L2	DNA binding	Yes	missense	non-functional	0.56 (0.97)	Negative	Negative	3	0	0	F44		A44
Epi 221	38	PR	255	7	ATC→**T**TC	Ile→Phe	AI		S9	DNA binding	No	missense	non-functional	0.15 (0.13)	Negative	Negative	3	2	0	R9	V	D24
Epi 257	50	PR	337	10	CGC→C**T**C	Arg→Leu	AI			Tetramerization	No	missense	non-functional	0.04 (0.04)	Positive	Positive	4	1	1	F36		A36
Epi 153	54	PR	175	5	CGC→C**A**C	Arg→His	AI		L2	DNA binding	Yes	missense	non-functional	4.9 (4.1)	Negative	Negative	3	0	0	F56		A56
Epi 196	55	PR	248	7	CGG→C**A**G	Arg→Gln	NI		L3/DNA	DNA binding	Yes	missense	non-functional	3.25 (3.6)	Positive	Positive	4	1	0	F48		A48
Epi 032	64	PR	337	10	CGC→**T**GC	Arg→Cys	AI			Tetramerization	No	missense	non-functional	0.06 (0.13)	Positive	Positive	4	0	0	F90		A90
Epi 037	61	PR	151	5	CCC→C**G**C	Pro→Arg	AI			DNA binding	No	missense	non-functional	0.07 (0.00)	Negative	Negative	3	0	0	F66		A66
Epi 087	47	PR	175	5	CGC→C**A**C	Arg→His	AI		L2	DNA binding	Yes	missense	non-functional	4.9 (4.1)	Positive	Positive	3	1	0	F56		A56
Epi 214	68	PR	193	6	CAT→C**T**T	His→Leu	AI		L2	DNA binding	Yes	missense	non-functional	0.20 (0.13)	Negative	Negative	3	1	0	F27		A27
Epi 015	53	SD	282	8	CGG→**T**GG	Arg→Trp	AI		H2	DNA binding	No	missense	non-functional	2.2 (0.97)	Positive	Positive	3	0	0	F52	SV	D72
Epi 177	57	SD	220	6	TAT→T**G**T	Tyr→Cys	AI			DNA binding	No	missense	non-functional	1.27 (1.7)	Negative	Negative	3	1	0	F15	LSV	D40
Epi 235	67	SD	205	6	TAT→**G**AT	Tyr→Asp	ND		S6	DNA binding	No	missense	non-functional	0.07 (0.09)	Negative	Negative	4	2	1	0	LV	D21
Epi 110	58	SD	273	8	CGT→**T**GT	Arg→Cys	ND		DNA	DNA binding	No	missense	non-functional	2.55 (1.1)	Negative	Negative	3	0	1	0	NA	D9
Epi 191	60	SD	127	5	TCC→T**T**C	Ser→Leu	AI			DNA binding	No	missense	non-functional	0.08 (0.04)	Negative	Negative	3	2	0	F52		A52
Epi 194	41	SD	244	7	GGC→G**A**C	Gly→Asp	ND		L3	DNA binding	Yes	missense	non-functional	0.24 (0.31)	Positive	Negative	3	1	0	0	LSV	D30
Epi 233	58	SD	255	7	ATC→A**G**C	Ile→Ser	AI		S9	DNA binding	No	missense	non-functional	0.04 (0.00)	Negative	Negative	3	1	0	F40		A40
Epi 063	67	SD	175	5	CGC→C**A**C	Arg→His	NI		L2	DNA binding	Yes	missense	non-functional	4.9 (4.1)	Negative	Negative	3	2	0	F15	V	A66
Epi 011	45	PD	213	6	CGA→**T**GA	Arg→Ter	No		L2/L3		Yes	nonsense	no data	1.05 (1.3)	Negative	Negative	3	0	0	F96		A96
Epi 095	29	PD	483–485°	5			AI	delCAT	L2		Yes	nonsense	no data		Negative	Negative	3	1	1	0	NA	D9
Epi 203	41	PD	175	5	CGC→C**A**C	Arg→His	AI		L2	DNA binding	Yes	missense	non-functional	4.9 (4.1)	Negative	Negative	3	1	1	0	NA	D9
Epi 002	52	PD	151	5	CCC→**T**CC	Pro→Ser	AI			DNA binding	No	missense	non-functional	0.36 (0.35)	Negative	Negative	3	2	0	F96		A96
Epi 215	61	PD	325	9	GGA→**T**GA	Gly→Ter	ND			Tetramerization	Yes**‡**	nonsense	no data	0.01 (0.04)	Negative	Negative	4	1	0	F24	V	A44

°, Nucleotide number; ^1^, The bolded bases indicate the base change; ^2^, Functional predictions derived from a computer model that takes into account the 3D structure of wild-type and mutant proteins and is trained on the trans activation dataset from Kato et al. Mutations are classified as “functional” or “non-functional”. More details here: http://www-p53.iarc.fr/Help.html#StructureClass; ^a^, Frequencies reported in IARC database (http://www.iarc.fr/p53/) release October 2006. The frequencies are based on a total of 22822 reported mutations in all type of cancer and in 2274 reported mutations in breast cancer (brackets); T N M, TNM-classification, AJCC 2002 = UICC 2002, T, size or direct of the primary tumor; N, spread to regional lymph nodes; M, distant metastasis; ˆ, “F” followed by a number indicates that the patient was free of disease at that number of months of follow-up. “R” followed by a number indicates that the patient was alive at that number of months of follow-up but had suffered a relapse; ®, Site of relapse L, Locoregional; S, Skeletal; V; Visceral; ^*^, “A” followed by a number indicates that the patient was alive at that number of months of follow-up. “D” followed by a number indicates that the patient died at that number of months of follow-up; ^‡^, Characterized as a mutation affecting L2/L3 domain, since it leads to truncation of the protein and will mostly affect L2/L3 domain; AI, Allelic imbalance; NA, Not available; ND, not done; NI, Not informative.

**Table 3:** Characteristics of *CHEK2* mutants found and clinical data

Patient	Age (Yrs)	Clinical response	Codon	Exon	Nucleotide change^1^	Amino acid change	LOH	Protein domain	Predicted mutation	EReceptor	PReceptor	T	N	M	Relapse-free Survival^3^	Site of relapse	Overall Survivalˆ
Epi 151	57	PR	364	9	ATA→A**C**A	Ile→Thr	NI	kinase domain	missense	Positive	Positive	3	1	0	F60		A60
Epi 203	41	PD	95	1	CGA→**T**GA	Arg→Ter	AI		nonsense	Negative	Negative	3	1	1	0	NA	D9
Epi 132	44	PD	95	1	CGA→**T**GA	Arg→Ter	AI		nonsense	Positive	Positive	5	2	0	*F60		A60

^1^, The bolded bases indicate the base change; T N M, TNM-classification, AJCC 2002 = UICC 2002, T, size or direct of the primary tumor; N, spread to regional lymph nodes; M, distant metastasis; ^3^, “F” followed by a number indicates that the patient was free of disease at that number of months of follow-up. “R” followed by a number indicates that the patient was alive at that number of months of follow-up but had suffered a relapse; ˆ, “A” followed by a number indicates that the patient was alive at that number of months of follow-up. “D” followed by a number indicates that the patient died at that number of months of follow-up; AI, Allelic imbalance; NI, Not informative; NA, Not available. “^*^” This patient subsequently relapsed with distant metastases at 64 months.

## Results

### *TP53* Mutations and Response to Therapy

The *TP53* mutations identified in the tumors of the patients treated with epirubicin together with the clinical response to therapy and follow-up data are presented in Table 2. Somatic *TP53* mutations were identified in 23 (21.5%) of the patients. Normal tissue (WBC) was available from 18 of these for germline characterization, revealing none of the mutations identified to be germline alterations. Of the 23 mutations detected, 20 were missense and 3 were nonsense. One mutation (del483CAT) has not been reported previously either in breast cancer or in any other tumor type (IARC database: http://www.iarc.fr/p53/). Twelve of the mutations directly or indirectly affected the L2/L3 domains of the p53 protein (Table 2) previous found to predict a poor prognosis [39] and drug resistance [14], [40]. For statistical comparison, mutation Gly325Ter (patient Epi215) located to the tetramerization domain is grouped together with the mutations affecting the L2/L3 domain, since this mutation leads to truncation of the protein and with loss of tetramerization and functional defects similar to L2/L3 mutations [41].

There was a statistical significant correlation between *TP53* mutation status and lack of treatment response (PD) (Table 4; p = 0.0358; Fisher exact test). When tumors harboring *TP53* mutations affecting the p53 L2/L3 DNA-binding domains were compared to those with wild-type *TP53* or *TP53* mutations outside the L2/L3 domains, this correlation was further strengthened (p = 0.0136).

**Table 4:** Clinical response in relation to different parameters

	Clinical response				Statistical significance	
	CR (n = 3)	PR (n = 50)	SD (n = 44)	PD (n = 10)	*P* ^1^	*P* ^2^
***TP53***						
Wild type (n = 84)	2	41	36	5		
All mutations (n = 23)	1	9	8	5	0.0358	0.0488
Mutations affecting L2/L3 (n = 12)	1	5	2	4	**0.0136**	0.0439
***CHEK2***						
Wildtype (n = 104)	3	49	44	8		
All mutations (n = 3)		1		2	**0.0226**	0.0631
***TP53+CHEK2*** *						
All mutations in *TP53*+*CHEK2*	1	10	8	6	0.0101	0.0183
Mutations affecting *TP53* L2/L3+*CHEK2*	1	6	2	5	**0.0032**	0.0165

*P^1^* with regard to clinical response comparing CR+PR+SD versus PD

*P^2^* with regard to clinical response comparing CR+PR versus PD

* One of the PD patients has got a mutation both in *CHEK2* and *TP53* (L2 domain), this has been taken into consideration under calculation of statistical significance

1 P, with regard to clinical response comparing CR+PR versus PD; ^2^P, with regard to clinical response comparing CR+PR+SD versus PD; ^*^, One of the PD patients has got a mutation both in *CHEK2* and *TP53* (L2 domain), this has been taken into consideration under calculation of statistical significance.

The previously described *TP53* polymorphism, Arg72Pro [42] was detected in 31 (29%) of our patients. No correlation was found between this polymorphism and lack of treatment response (p = 0.2750; Fisher exact test) or *TP53* mutational status (p = 0.2024).

### *CHEK2* Mutations and Response to Therapy

Table 3 presents the patients with detected *CHEK2* mutations together with a description of the clinical response and follow up-data. *CHEK2* mutations were identified in three out of the 109 patients (2.8%). Notably, each of the *CHEK2* mutations identified was also present in patient lymphocyte DNA, confirming a germline origin. The Arg95Ter (C283T) mutation is novel. This mutation was present in two patients (Epi132 and Epi203) living in different parts of Norway with no known family relationship. However, linkage analysis using microsatellite markers (D22S275, D22S272, D22S1172 and D22S423) suggested a common founder mutation (data not shown). The C283T transition generates a novel stop codon in exon 1 of *CHEK2*, leading to truncation of the Chk2 protein. LOH analysis indicated loss of the wild-type *CHEK2* allele in the both tumors from the two patients harboring this mutation (Epi132 and Epi203). Both these tumors were non-responsive to epirubicin therapy (PD). In contrast, the third patient with a germline *CHEK2* mutation (patient Epi151; point mutation at T1091C, Ile364Thr) had a partial response to epirubicin therapy. This tumor was non-informative with respect to LOH. Taking all *CHEK2* mutations together, they predicted resistance to epirubicin (p = 0.0226).

The previously described silent Glu84Glu (A252G) polymorphism [24], [43] in exon 1 was detected in two (1.9%) patients. No association between this polymorphism and treatment response was recorded.

One of the tumors (Epi203) harboring the C283T substitution (Arg95Ter) also harbored a somatic *TP53* mutation in codon 175, Arg175His, located in the L2 domain of p53 (Table 2). This mutation was detected in another four of our patients treated with epirubicin (Table 2). In addition, *TP53* Arg175His mutation was recorded in one patient of our previous study evaluating response to doxorubicin [13]. The fact that none of the Arg175His patients presented here or in our previous study revealed resistance to therapy (PD) suggests this mutation may not cause resistance to anthracyclines in breast cancers *in vivo*. Omitting the tumor harboring both a *CHEK2* and a *TP53* mutation (patient Epi203) from statistical analysis, Chk2 mutations (n = 2) were non-significantly associated with therapy resistance (p = 0.1633). In a previous study [26], however, we analyzed for *CHEK2* mutation status in relation to therapy outcome in a cohort of patients from doxorubicin study [13]. In that study [26], we detected the previously identified mutation Ile157Thr. In addition, we detected a novel nonsense somatic mutation (1368InsA). This mutation was associated with lack of function *in vitro*; moreover, it was associated with drug resistance *in vivo*. Analyzing our material and this cohort [26] together, (n = 160), *CHEK2* mutations (n = 5 in total) predicted for resistance to doxorubicin and epirubicin therapy (p = 0.0123). Even though, excluding patient Epi203 (harboring *TP53* Arg175His and Arg95Ter *CHEK2* mutation) as well as other patients harboring *TP53* L2/L3 mutations (n = 129), *CHEK2* mutations (n = 4 in total) predicted for resistance to doxorubicin and epirubicin therapy (p = 0.030).

### *TP53* and *CHEK2* Mutations Combined and Response to Therapy

Assuming that *TP53* and *CHEK2* mutations may substitute for each other, we analyzed for the predictive effect of mutations in both genes. The occurrence of a mutation affecting either *CHEK2* or *TP53* strongly predicted therapy resistance (*p* = 0.0101; Fisher exact test). When tumors harboring *TP53*-L2/L3 mutations and *CHEK2* mutations were compared with those wild-type or *TP*53 mutations outside the L2/L3 domain, the correlation was further strengthened (p = 0.0032; Fisher exact test). The significance was preserved when comparing patients with a PD to objective responders (CR and PR) excluding patients with stable disease (SD) from the statistical analysis (Table 4).

### *p14^(ARF)^* Mutations and Promoter Methylations

Neither mutations nor polymorphisms in the coding region of *p14^(ARF)^* were observed among the 107 patients analyzed. Likewise, no promoter methylations were detected.

### Influence of *CHEK2* and *TP53* Mutation Status on Relapse-Free Survival

**Figure 1:**
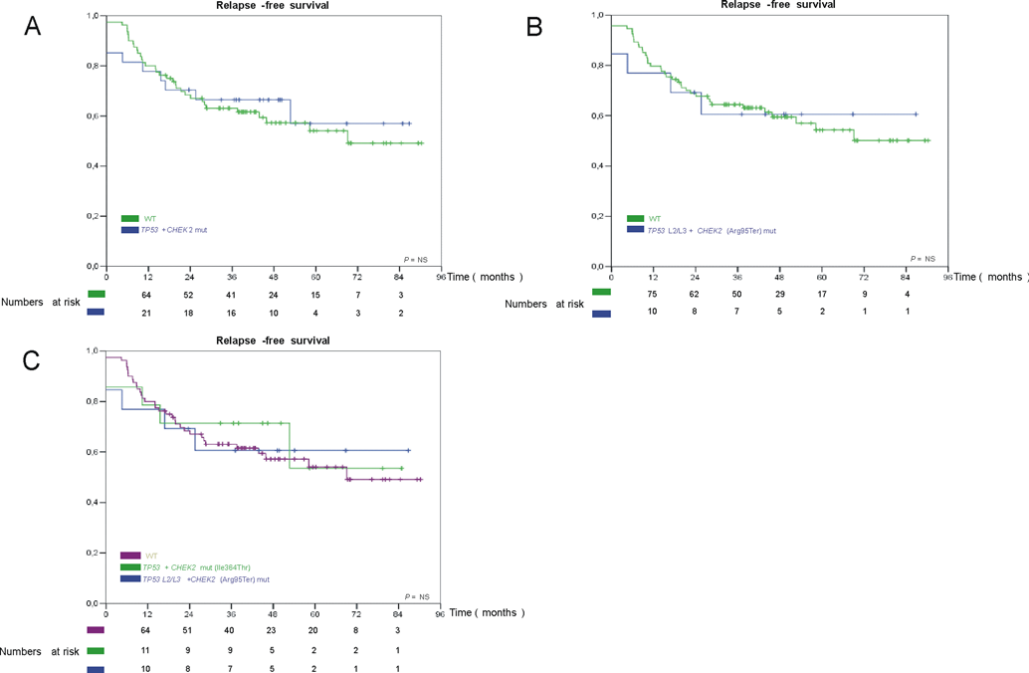
Kaplan-Meyer analysis of the relapse-free survival of the patients according to mutations. WT, wild-type; *TP53*+*CHEK2* mut, all found mutations in *TP53* and *CHEK2*; *TP53* L2/L3+*CHEK2* (Arg95Ter) mut, *TP53* mutations affection L2/L3 domain and *CHEK2* mutations affecting kinase function; *TP53*+*CHEK2* (Ile364Thr), mutations not affecting L2/L3 domains and *CHEK2* mutations not affecting kinase function. Deaths due to causes other than breast cancer are treated as censored observations. Each “+” mark represents the time one patient was censored. NS, Non significant.

Because of the limited time of the follow-up, no formal statistical assessment of overall survival was performed. Details regarding outcome for individual patients with mutations are described in Table 2 and 3 to make these data available to the reader. Relapse-free survival is depicted in (Figure 1). Figure 1A shows relapse-free survival for the patients with *TP53* and *CHEK2* mutations (all mutations found) compared to patients without any *TP53* or *CHEK2* mutations, no difference in relapse-free survival was observed. Similar, no difference was seen when grouping *TP53* mutations outside L2/L3 and *CHEK2* mutation not affecting kinase function (Ile364Thr) as wild-type (Figure 1B). Grouping tumors harboring a mutation in L2/L3 together with *CHEK2* mutations affecting kinase domain (Arg95Ter) in one group, mutations outside *TP53* L2/L3 and Ile364Thr as one group and tumors without any found mutations in *TP53* and *CHEK2* separately, again no noticeably difference in relapse-free survival were seen (Figure 1C). Notably, in addition to a short median follow-up time, a total of 35 patients with a sub-optimal response to epirubicin received subsequent treatment with paclitaxel, which may have influenced the outcome.

### *CHEK2* Mutant's Capability to Form Dimers

**Figure 2:**
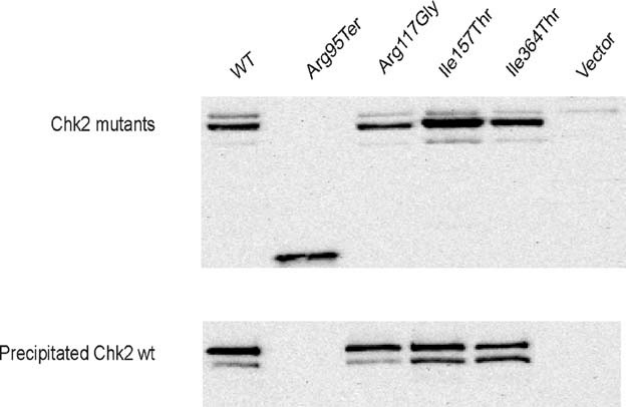
Pulldown-assay for *CHEK2* mutants. V5-tagged Chk2 mutants were co-expressed with Xpr-tagged wt-Chk2 in U-2-OS-cells and immunoprecipitation was performed using anti-V5 antibody. Expression of the Chk2 mutants was monitored by anti-V5 based Western blot analysis prior to immunoprecipitation (upper panel). The Chk2 mutant's ability to dimerize with the wild-type protein was detected by anti-Xpr Western blot analysis of the precipitate (lower panel).

To investigate whether the identified *CHEK2* mutations affect the ability of the Chk2 protein to form dimers, co-transfection and immunopresipitation of V5-tagged mutants and Xpress-tagged wild-type Chk2 were performed using *CHEK2* low-expressing U-2-OS cells. As we identified the previously characterized *CHEK2* germline mutants variants Arg117His (n = 2 and Ile157Thr (n = 1) among patients allocated to primary treatment with paclitaxel in our ongoing study, these mutants were evaluated together with Arg95Ter and Ile364Thr. The results presented in Figure 2 show that all Chk2 variants carrying a point mutation were able to form dimers with wild-type Chk2, whereas the Arg95Ter variant was not.

### Kinase Activity of *CHEK2* Mutants

To investigate whether the identified *CHEK2* mutants retained the wild-type kinase activity, an *in vitro* Chk2 kinase assay with respect to Chk2 autophosphorylation and Cdc25 substrate phosphorylation was performed. The U-2-OS cells were preferred for this assay because they were previously found to express only low levels of endogenous Chk2 [44]. This was confirmed by us using an antibody recognizing endogenous protein (data not shown). These cells have previously been used by other investigators to study Chk2 kinase activity [44], [45], [46].

**Figure 3:**
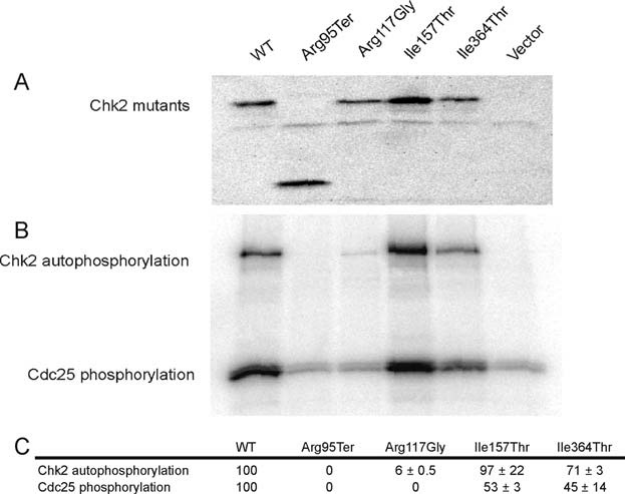
Kinase activity of *CHEK2* mutants. A) Level of Chk2 mutants immunoprecipitated from U-2-OS cells, used as input for kinase activity assay, monitored by anti-V5 based Western blot analysis. B) Autoradiogram showing *in vitro* kinase activity of Chk2 mutants with respect to both Chk2 autophosphorylation and Cdc25 phosphorylation. C) Kinase activity of *CHEK2* mutants normalized for kinase-input, based on band intensities in Figures 3A and B.

The two mutants Arg117Gly and Ile157Thr were previously tested for *in vitro* kinase activity [47], but were both included here, together with wild-type *CHEK2* as controls. Compared to wild-type Chk2, the Ile157Thr mutant retained wild-type kinase activity. The mutant Ile364Thr showed partially reduced kinase activity both in term of Cdc25-phosphorylation and autophosphorylation (Figure 3). In contrast, the mutant Arg117Gly showed strongly reduced kinase activity while the Arg95Ter mutant was totally devoid of any Chk2 kinase activity. The activity recorded for Ile157Thr and Arg117Gly was consistent with previously reported results for these two mutants [47]. Notably, there was an internal consistency with respect to percentage activity reduction comparing individual mutants with respect to autophosphorylation and phosphorylation of Cdc25 (Figure 3).

**Figure 4:**
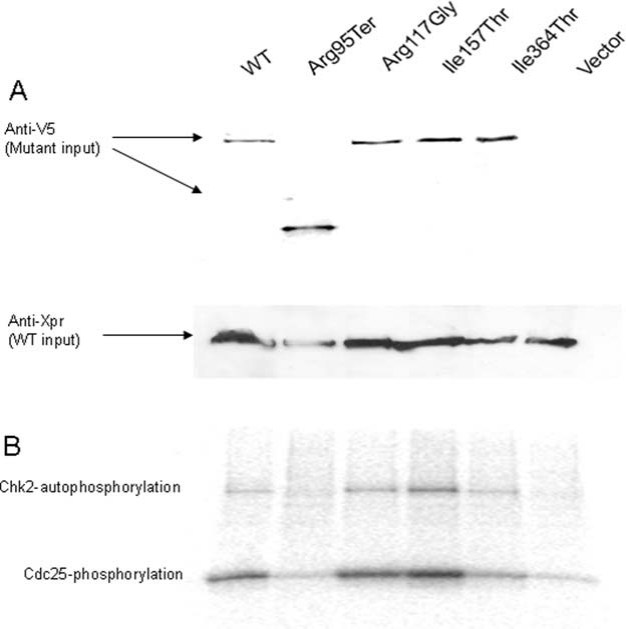
Kinase activity of *CHEK2* mutant's co-transfected with *CHEK2* wild-type. A) Kinase assay input of V5-tagged mutant Chk2 and Xpr-tagged wild-type Chk2, monitored by anti-V5 and anti-Xpr based Western blot analysis. B) Autoradiogram showing *in vitro* kinase activity (Chk2 autophosphorylation and Cdc25 phosphorylation) of Chk2 mutants with co-precipitated Chk2 wild-type.

Since enzymatically active Chk2 exists as dimers, it was important to determine the effect of Chk2 mutants on wild-type/mutant heterodimer kinase activity. The effect on Chk2 kinase activities (Chk2 autophosphorylation and Cdc25 substrate phosphorylation) of the individual mutants were therefore determined after co-transfection with wild-type Chk2 as described in Materials and Methods. The results from this co-transfection-kinase assay (Figure 4) were similar to those of the single-transfection assay (Figure 3) except in the case of the Arg117Gly mutant, which expressed a substantial kinase activity when complexed with wild-type Chk2. This is consistent with previous data indicating that the Arg117Gly mutant has neglectable kinase activity itself but dimerizes efficiently to Chk2 wild-type without strongly affecting the wild-type Chk2 activity. Hence, the activity detected is probably caused by the co-transfected and co-precipitated wild-type protein.

**Figure 5:**
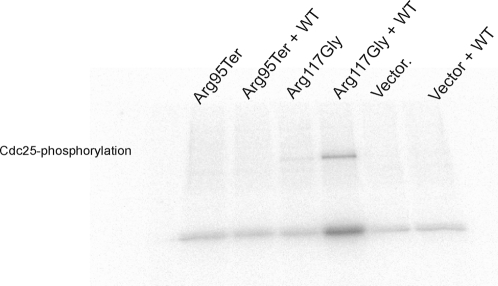
Contribution of co-precipitated Chk2 wild-type to the activity in the *in vitro* assays. Transfection of the Arg117Gly mutant with and without Chk2 wild-type, along with Arg95Ter +/− wild-type. Arg117Gly, when transfected alone, does not display higher kinase activity (Cdc25 phosphorylation) than Arg95Ter or negative control. This strongly indicates that the contribution of endogenous Chk2 is non-significant.

To rule out the possibility that endogenously expressed wild-type Chk2 contributed to observed Arg117Gly kinase activity shown in Figure 4, we compared the Arg117Gly variant activity in the presence or absence of co-transfected wild-type Chk2 to the activities of Arg95Ter under the same conditions. The Arg95Ter variant does not form dimers with wild-type Chk2. As seen in Figure 5, Arg117Gly, which forms dimers with Chk2 wild-type, allows increased activity when co-transfected with wild-type as compared to the corresponding activity for the Arg95Ter mutant. The fact that Arg117Gly, when transfected alone, displays very similar activity as Arg95Ter or negative control (background levels), strongly indicates that the contribution of endogenous Chk2, which, similarly to exogenously expressed wild-type Chk2 co-precipitate with Arg117Gly is non-significant.

### Family Cancer Incidence in Relation to *CHEK2* Germline Mutations

**Figure 6:**
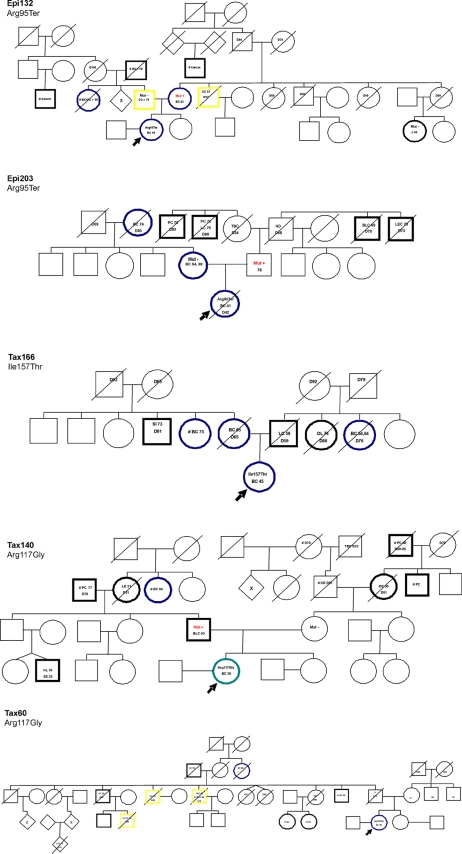
Pedigrees of the breast cancer cases with germline mutations in *CHEK2*. The index individuals initially screened are indicated with arrows. All cancer patients marked in bold, and cancers are indicated by type and age at diagnosis. D followed by number indicates the age of death. #, indicate that diagnosis could not be verified from medical documents. Mut −, indicates individuals tested negative for relevant mutations. Mut +, indicates that individuals hold the relevant mutation. The trees have been altered to preserve anonymity, but the meaning of the report is not affected by these alterations. BC, Breast cancer; BD, Blood disease; BLC, Bladder cancer; CC, Colon cancer; EC, Endometri cancer; GC, Gastric cancer; HD, Heart disease; HL, Hodgkins Lymphoma; L, Lymphoma; LAC, Larynx cancer; LC, Lung cancer; LE, Leukemia; LEC, Liver cancer; OC, Ovarian cancer; OL, Oral Lymphoma M; P, Parkinson; PC, Prostate cancer; SA, Sarcoma; SE, Seminom; SI, Carcinoid in small intestine; TBC, Tuberculosis.

Following an initial report of a family with a *CHEK2* germline mutation expressing an increased cancer incidence resembling the Li-Fraumeni syndrome [24], recent studies have revealed the more common *CHEK2* mutations to be associated with a moderately increased risk of breast and colorectal cancers. We hypothesized that *CHEK2* mutations having a detrimental effect on drug sensitivity could be associated with a more aggressive, Li-Fraumeni or a Li-Fraumeni-like (LFL) cancer syndrome [48]. Except from the patient harboring the Ile364Thr mutation who did not have any known congestion of cancer disease in the family, a detailed assessment of family cancer history was performed for each patient harboring a germline *CHEK2* mutation. The family cancer pedigrees are depicted in Figure 6.

While patients harboring *CHEK2* germline mutations revealed different types of cancers (mainly breast and tumors of the gastrointestinal area) in their family, surprisingly, no distinct pattern discriminating families harboring the Arg95Ter mutation from the other *CHEK2* mutated families could be identified. One of them (Epi203), who inherited the mutation from her father's side of the family, had no accumulation of either breast or colorectal cancer on that side. It should be noted, however, that two brothers of her fathers mother had prostate cancer, and two siblings of his father having hepatocellular carcinoma and bladder cancer, respectively), while the other expressed a disease pattern resembling what has been seen with the more common *CHEK2* mutations, like del1100C [25].

## Discussion

*TP53* plays a key role as a tumor suppressor gene. Its protein product activates processes such as growth arrest, DNA repair, apoptosis and/or senescence in response to genotoxic damage as well as oncogene activity [49], [50]. Despite being extensively studied, critical issues regarding regulation of the p53 protein remain poorly understood, and conflicting evidence obtained in different experimental systems make the clinical relevance of experimental data questionable.

Chemoresistance is the main obstacle to cancer cure in most malignancies, including breast cancer. Previously, we found *TP53* mutations affecting the L2/L3 DNA binding domain to be associated with lack of responsiveness to doxorubicin monotherapy [13] as well as mitomycin and 5-fluoro-uracil in concert [14]. However, some tumors revealed therapy resistance despite harboring wild-type *TP53*. Postulating that these tumors may harbor genetic disturbances in genes playing a key role in the p53 pathway, we here sequenced *TP53* along with *CHEK2* and *p14^(ARF)^*, the latter two known to play a critical role as p53 activators, in tumors from 109 patients treated with epirubicin monotherapy. Our results confirm *TP53* mutations, in particular those affecting the L2/L3 domains, to be associated with drug resistance. Most importantly, we also found *CHEK2* mutations generating a non-functional protein in our *in vitro* assays to be associated with drug resistance. In contrast, none of our tumors harbored either mutations or expressed promoter hypermethylations affecting the *p14*.

Based on *in vitro* assays, we were able to classify the different Chk2 mutants with respect to dimerization capability as well as kinase activity (Chk2 autophosphorylation and Cdc25 substrate phosphorylation). In addition, the kinase activities of the Chk2 wild-type/mutant complexes were monitored in co-transfection experiments. Notably, each point mutation (except for Arg117Gly) revealed similar relative kinase efficacy whether co-transfected with wild-type Chk2 or not (Figure 3 and 4). Cells co-transfected with Arg117Gly and wild-type Chk2 revealed kinase activity, probably due to the contribution of the wild type protein in Chk2 mutant – wild-type heterodimers. In contrast, cells transfected with Arg95Ter revealed no kinase activity whether co-transfected with wild-type Chk2 or not, clearly distinguishing this mutation from the others (Figure 3 and 5).

All *in vitro* assays were based on transfection of the U-2-OS cell line, a cell line known to express wild-type Chk2 at low levels, and previously used by other investigators to study Chk2 activity [44], [45], [46]. Since we were not able to obtain satisfactory technical quality of the kinase assay in cell lines negative for Chk2 (HCT 15 and HCT 116), we assessed potential background kinase activity due to endogenous Chk2 by performing western blot analysis revealing the endogenous levels of Chk2 in U-2-OS cells to be non-significant compared to the exogenously expressed Chk2 levels (data not shown). We also performed a separate kinase assay, directly comparing the effect of binding partners for the dimerizing Arg117Gly and the non-dimerizing Arg95Ter. This assay also revealed the contribution of endogenous Chk2 to be non-significant (Figure 5).

Taking our *in vitro* findings together with *in vivo* observations, our present data confirm that the functionally defective *CHEK2* Arg95Ter mutation, together with LOH, is associated with resistance to anthracycline therapy. In contrast, the patient harboring the Ile364Thr mutation, moderately reducing phosphorylation activity, responded well to therapy. The other missense mutations; Arg117Gly and Ile157Thr were observed among patients receiving paclitaxel therapy only; thus, their influence on anthracycline sensitivity *in vivo* could not be addressed. Yet, based on the finding that the Arg117Gly mutant expressed no intrinsic activity, but readily dimerized to the wild-type protein without abolishing its activity, we hypothesize that this mutation and, probably, other yet unidentified *CHEK2* mutations with a similar lack of intrinsic kinase activity, may cause resistance to anthracycline therapy if combined with LOH in breast cancer.

Our present findings have two major implications. First, we confirm that mutations in genes encoding proteins located within the same functional pathway may substitute for each other with respect to drug sensitivity, revealing for the first time a functional pathway critical to chemotherapy response *in vivo*. Second, the identification of mutations in the *CHEK2* but not in the *p14^(ARF)^* gene in resistant tumors suggests that Chk2 mediated phosphorylation of p53 is a critical event in executing anti-tumor effect as a response to DNA damaging agents in breast cancer. This adds to our understanding not only of the function of p53 but Chk2 as well. p53 undergoes phosphorylation at multiple sites by different kinases, including Chk2 [51]. While activation of the ATM leading to direct (Ser 15) and Chk2-mediated (Ser 20) phosphorylation of p53 is considered an important mechanism for triggering p53 activation in response to DNA damage [52], some reports suggest ATM [53] and even Chk2 [23] to be redundant to this function. Importantly, Chk2 has been shown capable of inducing ATM-independent apoptosis *in vitro* [21]. While Chk2 phosphorylates p53 at Ser 20, thereby stabilizing p53 by preventing MDM2 binding [19], Chk2 also phosphorylates p53 at six additional sites, including Ser 313 and Ser 314 located in the nuclear localization signal domain of p53 [51]. In addition, Chk2 phosphorylates other important targets like BRCA1, Cdc25A and Cdc25C involved in DNA repair, G1 and G2 arrest, respectively [54]. Despite the wide range of known Chk2 substrates relevant for DNA repair and cell cycle control, our present findings that *CHEK2* mutations leading to non-functional Chk2 protein may substitute for p53 mutations strongly advocate a role for Chk2 with respect to drug sensitivity executed through p53 activation.

Notably, one of the tumors (Epi203) with the Arg95Ter *CHEK2* mutation in addition harbored a somatic *TP53* mutation, Arg175His, with allelic imbalance for the *TP53* gene (Table 2). Importantly, among another four patients in this study (Epi063, Epi071, Epi087, Epi153) and one patient from our previous doxorubicin protocol [13] harboring the Arg175His mutation together with allelic imbalance for *TP53*, all five of these patients responded to anthracycline therapy either with a partial response or stable disease. In contrast, Epi132 and the only patient for whom we previously identified a non-functional *CHEK2* mutation (1368InsA; coding for a non-functional protein translate with cytoplasmic location [26]) expressed resistance to epirubicin and doxorubicin, respectively. Arg175His is a p53 “hot-spot” structural mutation reported to have defects with respect to transcriptional activation and also to negatively interact with wild-type p53 [55]. While this mutation has been shown to enhance chemoresistance upon transfection into p53 null Saos-2 cells [56], these osteosarcoma-derived cells may not necessarily be representative for breast cancers *in vivo*. Recent evidence strongly support p53 to be involved also in non-transcriptional mediated apoptosis by interacting with the Bcl-2/Bax system [57], and transcription-defect structural p53 mutants have been shown to execute non-transcriptional apoptosis in experimental systems [58]. Concomitant inactivation of Chk2 and p53 in breast cancer has been recorded by others [59], and the finding that a somatic mutation may generate a “growth advantage” in tumor cells already harboring a germline *CHEK2* mutation may not implicate an effect on drug sensitivity in tumors not yet exposed to cytotoxic compounds. Rather, it may indicate a growth advantage, probably related to loss of p21 function. Notably, in a previous study we found the p21 polymorphism G251A to be associated with an increased risk of developing large breast cancers but to have no effect on drug sensitivity [60], indicating that growth rate and drug resistance may be regulated independently. Taken together, we believe our findings advocate a role for Chk2 in executing cellular response to anthracycline-induced DNA damage.

As mentioned above, removing *TP53* mutated tumors including the double-mutated Epi203 from statistical analysis, *CHEK2* mutation status still predicted for resistance to anthracycline therapy. In addition, removing the tumors harboring the Arg175His mutation from the p53 “L2/L3” group strengthened the correlation to lack of treatment response to epirubicin (p = 0.0005).

Comparing the effects of mutations in the *CHEK2* gene to *TP53* mutations indirectly underlines the importance of the role of Chk2 to chemoresistance. Our present findings as well as results from our previous studies [13], [14] revealed that about 50% of the patients with tumors harboring *TP53* L2/L3 mutations to be non-responders to primary therapy. In contrast, all our three patients harboring a non-functional *CHEK2* mutation (the two Arg95Ter mutated patients here and our previous patient harboring the 1368InsA) expressed primary resistance to therapy. We previously hypothesized that therapy response in tumors harboring *TP53* L2/L3 mutations could be due to redundant pathways acting in concert [3]. Although no definite conclusion should be drawn from a limited number of observation, the fact that Chk2 not only phosphorylates p53 but also phosphorylates other substrates such as Cdc25A and Cdc25C [54] and E2F1 in response to etoposide-induced DNA damage [61] may indicate that inactivation of redundant pathways could take place in parallel.

The literature remains inconsistent with respect to whether the border amino acids 163, 195, 236 and 251 should be included in the p53 L2 and L3 domains [12]. Taking a conservative approach, we classified patient Epi56, harboring a mutation in codon 163, as a L2/L3 mutant. The patient harboring this mutation responded to therapy (PR). If this mutation was classified as outside the L2 domain, our p-value had been strengthened from *p* = 0.0136 to *p* = 0.0096.

Germline mutations in *TP53* cause the Li-Fraumeni and Li-Fraumeni-like cancer disposition syndromes. However, while the germline and somatic mutations associated with these syndromes reveal a preference for the same codons [48], *TP53* mutations affecting the DNA-binding domains seem associated with a poor prognosis [62], [63], [64] and, in particular, drug resistance [14], [40] in breast cancer. Thus, tumor suppression and tumor cell response to chemotherapeutics may involve different parts of p53 protein function. Following an initial report identifying a *CHEK2* mutation in a family expressing characteristics of the Li-Fraumeni syndrome [65], recent evidence has linked *CHEK2* founder mutations to a moderately increased risk of breast- and colorectal cancers with some additional disposition for other malignancies as well [66]. However, cancer incidence and phenotypes did not reveal an aggressive Li-Fraumeni or Li-Fraumeni-like tumor pattern. Similar to the two patients in our paclitaxel treatment arm harboring the rare but previously characterized mutation Arg117Gly and the patient with the Ile157Thr mutation, they expressed a moderately increased risk of breast and gastrointestinal cancers (Fig. 6). Thus, *CHEK2* resembles *TP53* in as much as there seems to be no direct correlation between effects of individual mutations with respect to tumor suppression and drug resistance.

Our finding that *TP53* mutations located to the DNA-binding domains predicts drug resistance may indicate transcriptional mechanisms to be involved in drug-induced cell death. p53 induced apoptosis has been associated with transcriptional induction of genes including *Puma* and *Noxa* as well as *Bax* in experimental systems [55], [67], [68]. Yet, recent evidence has revealed p53 to induce apoptosis through non-transcriptional mechanisms by direct protein interactions with members of the Bcl-2/Bax system and mitochondrial release of cytochrom c [57], [69]. In deed, there is evidence that the DNA-binding domains, in particular the L3 part of the protein, may be critical also to transcriptional-independent apoptosis [70]. Of particular note is the finding that Chk2 may regulate transcriptional-independent p53-mediated apoptosis in response to DNA-damage created through ionizing irradiation [71]. Interestingly, Krajewski et al [72] reported low expression of Bax assessed by immunostaining to be associated with a low response to chemotherapy in metastatic breast cancer. Although no conclusion should be drawn at this stage, together these findings are consistent with the challenging hypothesis that transcription-independent activation of Bax following Chk2-phosphorylation may represent a key pathway in p53 dependent cell death in breast cancer *in vivo*.

p14 acts by releasing p53 from MDM2 binding, and has been related to oncogene-induced p53 activation [73]. Recently, p14 was shown to affect p53 by additional mechanisms, including acetylations [74], response to ionizing radiation in human fibroblasts [75], and tumor-suppression following ionizing radiation in mice [76], [77]. These findings further links the retinoblastoma and p53 pathways [28]. As such, we believe the negative finding with respect to its role in chemoresistance adds important information.

Contrasting earlier findings by us and others [15], a recent study revealed *TP53* mutations to be associated with increased likelihood of having a complete response to chemotherapy [16]. These results may not necessarily be at conflict. In the latter study, patients received treatment with a “dose-dense” chemotherapy regimen; if confirmed, the combined data may outline a therapeutic indication for aggressive dose-dense therapy based on tumor *TP53*/*CHEK2* status.

So far attempts to identify single markers and, more recently, gene expression arrays predicting chemoresistance have not proved successful (see refs in [9], [10]). The findings presented here reveal for the first time defects in a functional gene cascade to be associated with drug resistance in a human cancer *in vivo*. Moreover, the findings are made in breast cancer, the most frequent malignant disease among women in the industrialized world, and relate to resistance to anthracyclines, the type of cytotoxic compounds most frequently employed for this malignancy.

While the only study we are aware of comparing *TP53* mutation status in primaries and their distant metastases suggested an increasing fraction of tumors to express mutated *TP53* during progression [78], we do not know the potential contribution of either *TP53* or *CHEK2* mutations to drug resistance in micrometastases or in metastatic disease. Yet the finding that one of our non-functional *CHEK2* mutations associated with chemoresistance (1368InsA) occurred as a somatic, not germline mutation, suggest such mutations may be selected for during tumor progression. We propose the findings presented here provide important beacons identifying a functional pathway [3] likely to be disturbed through different mechanisms in relation to therapy resistance in advanced disease.

In conclusion, we believe our findings here that mutations in the *TP53* and *CHEK2* genes each may cause resistance to anthracycline therapy in primary tumors to have wide implications to future research in this area. While results from experimental systems are mandatory generating hypotheses, conflicting data from *in vitro* studies underlines the pivotal role of identifying defects associated with therapy resistance *in vivo*. Either through mutations of the genes themselves, or inactivation of this functional cascade through co-factors, we believe identification of the Chk2 – p53 axis as critical to anthracycline therapy response provides a functional clue for further investigations in this area.
